# An Artificial Crown

**Published:** 1887-11

**Authors:** A. W. M'Candless

**Affiliations:** Davenport, Iowa.


ARTICLE X.

### AN ARTIFICIAL CROWN.

A. W. M'CANDLESS, D. D. S., DAVENPORT, IOWA.

The crown I am about to describe is not altogether original with me, but as I have seen no notice of it in any of the journals, I will try to give an idea of the manner in which it is constructed.

An ordinary plain tooth—the kind used in rubber work is as good as a plate tooth and usually much more easily obtained—suitable for the case in hand is selected and fitted as accurately as may be to the root which has been previously prepared, care having been taken that the root is cut quite a little above the margin of the gum.

The tooth is then backed with platina which should be allowed to extend down below the incising edge of the tooth.

A copper wire 18 guage—platina is preferred—and about 1½ inches in length is laid with the centre over the pins of the tooth, bent down around the outside of the pins and up between them, thus forming a loop securely holding the pins. The ends of the wire are now twisted together forming a pivot of the very best shape for secure fastening in the root. Enough cement is used to simply catch the end of the pivot when the artificial crown is in place.

When the cement is sufficiently hard to hold the pivot firmly, fill the remainder of the root with amalgam which must also extend over the entire surface of the backing. Thus the metals all become thoroughly amalgamated into one solid mass, making a secure permanent operation, leaving no cement exposed to the fluids of the mouth, to become disintegrated.

I have put on many of these crowns where any other crown seemed impracticable and impossible on account of the poor condition of the roots.

I recently attached a cuspid crown of this kind to a bicuspid root that seemed almost worthless, as there was little left of it and the crown having been absent for so long that the lower bicuspid came almost to the gum surrounding the upper root.

The shape of the root can well be imagined being quite depressed in the centre so that it would be impossible to perfectly ferrule it, whereas the amalgam could be thoroughly adapted to it.

This being a first bicuspid and my patient a young lady with otherwise pretty teeth, of course the adaptation of a perfectly natural looking crown in such a position was a gratification to both my patient and myself.

As I said before, I have placed many of these crowns on roots that seemed too far gone for any other good crown with which I am familiar and I have the first failure to hear from.—*Archixes of Dentistry.*

